# Coarse-Grained/Molecular Mechanics of the TAS2R38 Bitter Taste Receptor: Experimentally-Validated Detailed Structural Prediction of Agonist Binding

**DOI:** 10.1371/journal.pone.0064675

**Published:** 2013-05-31

**Authors:** Alessandro Marchiori, Luciana Capece, Alejandro Giorgetti, Paolo Gasparini, Maik Behrens, Paolo Carloni, Wolfgang Meyerhof

**Affiliations:** 1 International School for Advanced Studies (SISSA-ISAS), Neuroscience Sector, Trieste, Italy; 2 International Centre for Genetic Engineering and Biotechnology, Trieste, Italy; 3 Computational Biophysics, German Research School for Simulation Sciences, Juelich, Germany; 4 Department of Biotechnology, University of Verona, Verona, Italy; 5 Institute for Maternal and Child Health - IRCCS “Burlo Garofolo”, Trieste, Italy; 6 Department of Molecular Genetics, German Institute of Human Nutrition Potsdam-Rehbruecke (DIfE), Nuthetal, Germany; 7 Institute for Advanced Simulation IAS-5, Computational Biomedicine, Forschungszentrum Juelich, Juelich, Germany; University of Tokyo, Japan

## Abstract

Bitter molecules in humans are detected by ∼25 G protein-coupled receptors (GPCRs). The lack of atomic resolution structure for any of them is complicating an in depth understanding of the molecular mechanisms underlying bitter taste perception. Here, we investigate the molecular determinants of the interaction of the TAS2R38 bitter taste receptor with its agonists phenylthiocarbamide (PTC) and propylthiouracil (PROP). We use the recently developed hybrid Molecular Mechanics/Coarse Grained (MM/CG) method tailored specifically for GPCRs. The method, through an extensive exploration of the conformational space in the binding pocket, allows the identification of several residues important for agonist binding that would have been very difficult to capture from the standard bioinformatics/docking approach. Our calculations suggest that both agonists bind to Asn103, Phe197, Phe264 and Trp201, whilst they do not interact with the so-called extra cellular loop 2, involved in cis-retinal binding in the GPCR rhodopsin. These predictions are consistent with data sets based on more than 20 site-directed mutagenesis and functional calcium imaging experiments of TAS2R38. The method could be readily used for other GPCRs for which experimental information is currently lacking.

## Introduction

Bitter taste perception prevents humans and other mammals from ingesting toxic substances. The perception stems from the binding of bitter molecules to ∼25 specific G protein-coupled receptors (GPCRs) referred to as taste 2 receptors (TAS2Rs) [1], [2] (Fig. S1). TAS2Rs are located in special subsets of taste receptor cells [1]–[4]. They are able to detect multiple and diverse natural and synthetic organic molecules [5].

The most widely characterized bitter receptor at the genetic level is TAS2R38 [6], [7]. Single nucleotide polymorphisms in the *TAS2R38* gene (GenBank: AY258597.1) cause “blindness” to its agonists phenylthiocarbamide (PTC) and propylthiouracil (PROP) (Chart S1) [6]. This constitutes a well-characterized human genetic trait [7]. Indeed, in normal population, regardless of race, age and gender, there are many subjects that were able to perceive phenylthiocarbamide (PTC) and its related compounds, with the N-C = S moiety, while many other subjects do not [8].

Experimental 3D structural information on TAS2R38, as on any other bitter taste receptor, is lacking. Functional assays-validated bioinformatics approaches, complemented with molecular docking [9], have provided structural insights on agonist/TAS2R38 interactions. A similar procedure has been used successfully for other TAS2Rs [10]–[13]. The responses of the different receptor mutants have been measured upon application of increasing concentrations of agonists. If the EC_50_ value is larger than that of the wild-type (WT), the receptor sensitivity is impaired, whilst the contrary is true if the EC_50_ is lower. Higher maximal signal amplitude usually may stand for improved receptor activation relative to WT, whilst a lower one stands for an impaired activation. These pieces of information have been included in the model, providing insights in structure/function relationships. In spite of these insights, the approach has clear limitations in describing the active site cavity of the receptor. Structural predictions of TAS2R38 (as of any other TAS2R) is difficult because it shares a sequence identity with structural templates of less than 20% [9]. Hence, the orientation of the side-chains in the active site cavity is likely not to be correct.

**Figure 1:**
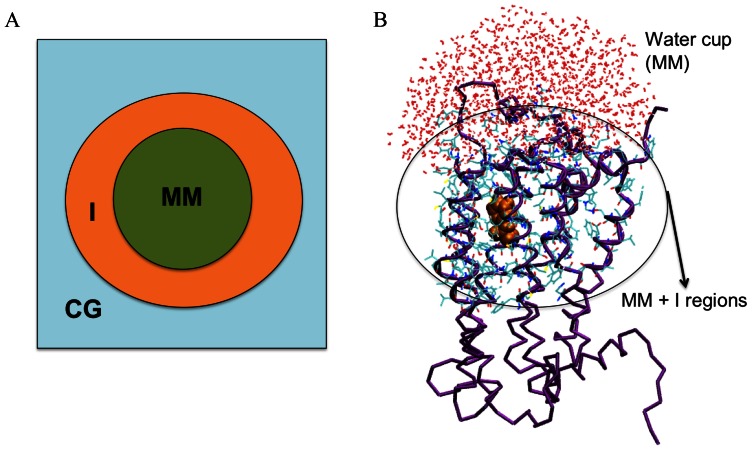
Molecular Mechanics/Coarse-grained system set-up. **A)** Schematic representation of the regions defined in the MM/CG model. The MM, I and CG regions are colored in green, orange, light blue, respectively. **B)** MM/CG representation of the hTAS2R38 receptor in complex with PTC. Water molecules and residues belonging to the MM and I regions are represented as lines. The agonist atoms are represented as orange spheres. The protein Cα atoms are represented in violet.

Recently, we have developed a combined atomistic-coarse grained approach [14] for structural predictions of agonist- and antagonist- GPCR complexes, the Molecular Mechanics/Coarse-Grained (MM/CG) molecular dynamics [15], [16]. Here, the ligand, the solvent surrounding it and the binding cavity are represented with an atomistic force field, while the rest of the protein frame is described using a Go-like [17] CG representation (Fig. 1A). The hydration is taken into account by including a sphere of water molecules centred on the ligand (Fig. 1B). An interface (I region) is defined between MM and CG regions that bridge the two different resolution models. In this way, the number of degrees of freedom is drastically reduced by up to 2 orders of magnitude [14]. This allows the system to be equilibrated in much shorter time scale, and it may be able to avoid artefacts caused by wrong orientations of side-chains in loop and in helices far from the active site. We have shown that 0.8 µs MM/CG simulations of a GPCR/inverse-agonist complex for which the 3D X-ray structure is experimentally available -the β2 adrenergic receptor/S-Carazolol (β2 AR/S-Car)- could reproduce the results of full atomistic MD [14], [18]. Importantly, the approach turns out to recover the orientation of the S-Car ligand as in the X-ray structure irrespectively of its initial orientation [14]. The MM/CG simulation took less than a week on a 64 cpus PC cluster.

Here, we explore the capability of this approach to predict structural determinants of TAS2R38. First, we show that µs-long MM/CG simulations are capable to reproduce the structure of the β2 AR/S-Car complex X-ray structure starting from a homology model. The same setup applied to TAS2R38 structure in complex with PTC and PROP turns out to be fairly consistent with as many as 22 receptor mutants expressed for this work and tested for two different agonists, providing insights on the interaction between a bitter taste receptor and its agonists at an unprecedented level of accuracy.

## Results

### Validation of the MM/CG Method for Homology Models

**Figure 2:**
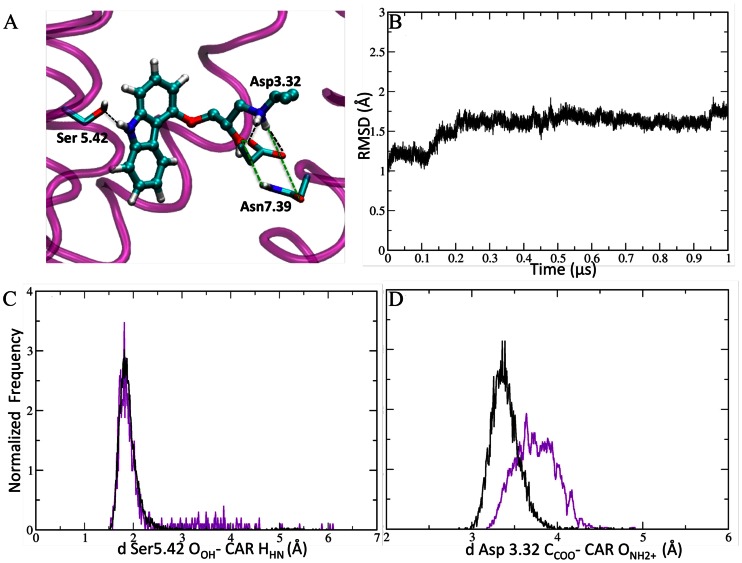
β2 AR in complex with the S-Car ligand. **A)** S-Car binding site of the central structure of the only cluster of the MM/CG simulations. This cluster represent 100% of the conformations of the adduct. **B)** RMSD of the Cα atoms plotted as a function of time in the MM/CG simulations. **C–D)** Distribution of agonist-protein H-bonds in the MM/CG simulations of the β2 AR/S-Car complex based on the X-ray structure [14] (black line) and on an homology model (violet line, this work).

We have recently shown [14] that MM/CG calculations of β2 AR in complex with its ligand S-Car are in agreement with the corresponding ones with all-atom MD on the same system [18]. The calculations are based on the X-ray structure of the complex [19]. Here we further investigate the predicting power of the MM/CG method using a homology model of the same complex (Fig. 2A). The chosen template for building up the model of the β2 AR was the structure of squid rhodopsin (PDB id 2z73) [20]. It displays a sequence identity of 20% with the target protein. This value is within the range of the identities between the hTAS2R38 and its best templates. After 0.8 µs, the β2 AR structure in complex with S-Car is similar to the X-ray structure (RMSD of the Cα atoms 2 Å, Fig. 2B). The interactions observed between the ligand and the protein present in the X-ray structure are reproduced with Ser5.42 and Asp3.32 side-chains forming H-bonds with the agonists amino groups, and Asn7.39 present in the ligand binding cavity (Fig. 2C–D) as it is in the X-ray structure. Hence, MM/CG simulations on a homology-modelled structure reproduce the ligand pose as in the X-ray structure [19], indicating that our approach can be used in general for ligand/GPCR's complexes. The same procedure is next applied to the human TAS2R38 receptor.

### Bioinformatics- and MM/CG-based Structural Predictions of TAS2R38

**Figure 3:**
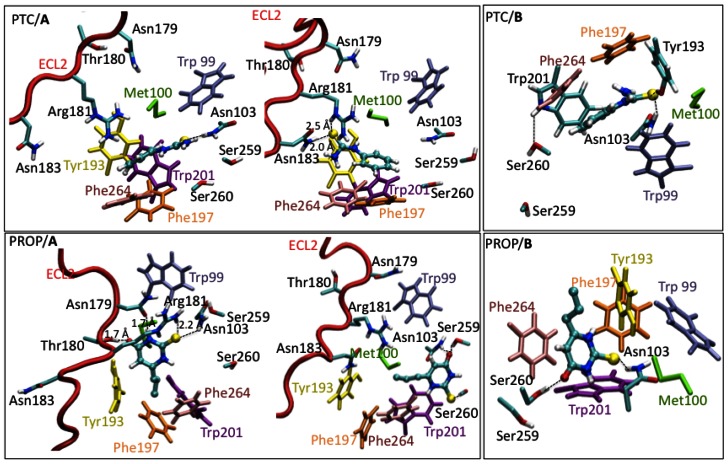
Central structures of PTC/A - PROP/A and PTC/B - PROP/B main clusters emerging from our MM/CG simulations. Residues forming mostly or exclusively hydrophobic contacts with the agonists (Met100, Phe264, Phe197, Trp99, Trp201, Tyr193) are colored in green, pink, orange, light violet, purple, yellow, respectively. The agonists are shown in ball-and-sticks representation and they are colored by atom type. The ECL2 loop interacts with the binding site only in the A complexes. It is shown in red cartoon. Selected distances for Asn179, Thr180, Arg181 and Asn183 residues in the ECL2 loop are shown for PTC/A and PROP/A.

**Figure 4:**
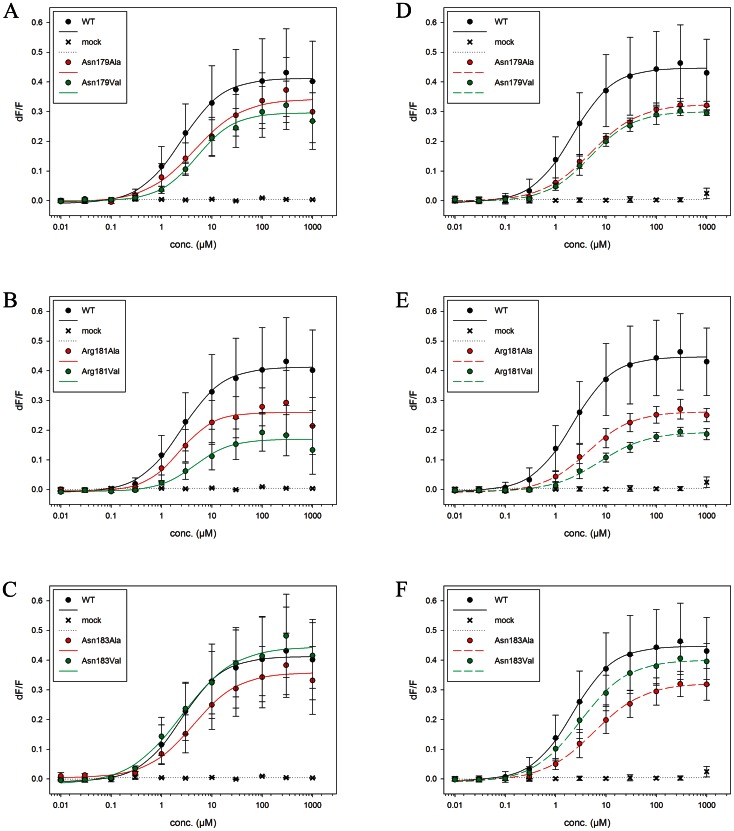
Dose-response curves. Dose-response curves of TAS2R38 wild type and mutants after stimulation with increasing PTC and PROP concentrations (0 to 1000 µM). Each point corresponds to the mean ± standard deviation. The mean is calculated from at least three independent experiments. **A–C)** PTC application, **D–F)** PROP application.

The predicted structural determinants of the protein, obtained by homology modelling, are either the same as those of ref. [9] (model **A**) or they are very similar except that they differ for the ECL2 loop (model **B**, selected ligands-receptor distances in Table 1). The latter points away from the binding cavity in **B**, resembling the ‘open’ conformations found in the non-rhodopsin templates (see Table S1). In contrast, in **A**, the ECL2 loop assumes a conformation close to the putative binding cavity (The rest of the folding domain is very similar to all the structures used as templates). The volume of the cavities can be appreciated in the Text S1 document and in Figure S3A and B. The structures of the corresponding adducts with PTC and PROP (details in Text S1), obtained by molecular docking (PTC/**A**, PTC/**B**, PROP/**A** and PROP/**B** hereafter), underwent two different runs of 0.6 µs each of MM/CG simulations [14] at room temperature. All adducts appear to be equilibrated after ∼0.1 µs, as shown by a plot of the RMSD of the Cα atoms and of the agonists atoms as a function of simulated time (Fig. S2). Although loop structure predictions are well known not to be very accurate [21], we conclude that ECL2 points away from the binding cavity in PTC/**B** and PROP/**B** complexes, and close to it in PTC/**A** and PROP/**A**. The PTC/**A** structure shows the ECL2 is close to the binding cavity, while in PTC/**B** the ECL2 is located away from it and in contact with the extracellular region. Three ECL2 residues, Asn179, Arg181 and Asn183 form either direct contacts with the agonists or contribute to shape the binding cavity in **A** but not in **B** (Fig. 3). Specifically, at times Arg181, Asn183 interact with PTC sulphur atom, Asn179 interacts with PROP NH group, Thr180 with PROP oxygen atom, and Arg181 with PROP sulphur atom (Fig. 3). This allows us to discriminate between the models with the conformation **A** from those with the conformation **B**. Indeed, we can predict that mutations such as Asn179Ala/Val, Arg181Ala/Val and Asn183Ala/Val do not affect agonist binding in PROP/**B** and PTC/**B** complexes, whilst they will in the case of PTC/**A** and PROP/**A**. Here we performed functional assays on these mutant variants following ref. [9]. The EC_50_ values of the mutants turn out to be similar to those of WT (Fig. 4A–F and Table 2). This leads us to discard the **A** models (The **A** models turn out not to be consistent with several other mutations performed by us - data not shown, then rhodopsin-like models were not considered). Thus, from now on therefore we consider only the PROP/**B** and PTC/**B** complexes.

**Table 1:** Selected MM/CG distances (in Angstrom) in the central structure of the main clusters of the PTC/B and PROP/B adducts.

Distance	PTC/B
Asn103-NH2 – PTC-S	3.1
Ser260-OH – Trp201-HN	2.5
Tyr193-HO – PTC-S	1.9
Phe197_Ring_ – PTC_Ring_	6.5
Phe264_Ring_ – PTC_Ring_	3.4
Trp201_Ring_ – PTC_Ring_	5.5
Trp99_Ring_ – PTC_Ring_	6.1
Met100_side chain_ – PTC_ring_	8.9
**Distance**	**PROP/B**
Asn103-NH2 - PROP-S	3.0
Ser260-HO – PROP-O	1.6
Phe197_ Ring_ – PROP_Ring_	6.3
Phe264 _Ring_ – PROP_Ring_	4.5
Trp201_ Ring_ – PROP_Ring_	5.2
Trp99_ Ring_ – PROP_Ring_	8.3
Met100_side chain_ – PROP_ring_	7.1

**Table 2:** Experimental EC_50_ and maximum activity values towards PTC and PROP obtained for WT TAS2R38 and selected mutants.

Variant	Agonist			
	PTC		PROP	
	EC_50_ (uM)	Max act	EC_50_ (uM)	Max act
WT	2.5 (**3**)	0.43 (**0.47**)	2.17	0.44
**Trp99**Ala	1.2 (**4.25**)	0.14 (**0.25**)	1.8	0.59
**Trp99**Val	1.8 (**2.7**)	0.28 (**1.12**)	5∼	0.93
**Met100**Ala	4.1 (**3**)	0.72 (**1.01**)	1.2	0.77
**Met100**Val	21.2* (**10**)	0.51 (**0.79**)	1.8	0.42
**Asn103**Ala	6.6* (**8**)	0.21 (**0.38**)	8.7*	0.65
**Asn103**Val	6.9* (**15**)	0.09 (**0.09**)	9.1*	0.41
**Asn103**Asp	–	0.06	23.8*	0.13
**Asn179**Ala	4.4	0.34	4.9	0.32
**Asn179**Val	4.9	0.29	5	0.30
**Arg181**Ala	2.2	0.26	4.3	0.26
**Arg181**Val	4.5	0.17	7.5	0.19
**Asn183**Ala	4.2	0.36	5.3	0.32
**Asn183**Val	2.5	0.44	3.1	0.40
**Phe197**Val	4.3	0.06	9.9*	0.12
**Trp201**Leu	–	0.25	–	0.02
**Trp201**Phe	21*	0.14	–	0.05
**Ser259**Ala	5.7 (**5.4**)	0.55 (**0.42**)	2.9	0.45
**Ser259**Val	99* (**27**)	0.02 (**0.04**)	21.8*	0.18
**Ser260**Ala	1.41	0.21	1.14	0.45
**Ser260**Val	9.8*	0.03	6.8*	0.09
**Phe264**Ala	–	0,06	–	0,06
**Phe264**Val	12.4*	0.06	25.9*	0.24

* Value statistically different from WT ∼ Estimated value, curve close to saturation. WT and the mutants already investigated in ref. [9] are in bold-face. For those, we report the values of ref. [9] in brackets.

### Models Validation

In PTC/**B**, the agonist sulphur atom forms H-bonds with Asn103 side-chain (Table 1 and Fig. 3). It also forms an H-bond with Tyr193 side-chain. Ser260 side-chain forms H-bonds to Trp201 side-chain. The PTC ring forms hydrophobic interactions with Phe197, with Trp201 and Phe264. In PROP/**B**, the agonist sulphur atom forms H-bonds to Asn103 NH_2_ group. The agonist oxygen atom forms H-bond to Ser260 side-chain. The PROP ring forms hydrophobic contacts with Trp201, Phe264 and Phe197. These hydrophobic residues, together with Tyr193, shape the binding cavity.

**Figure 5:**
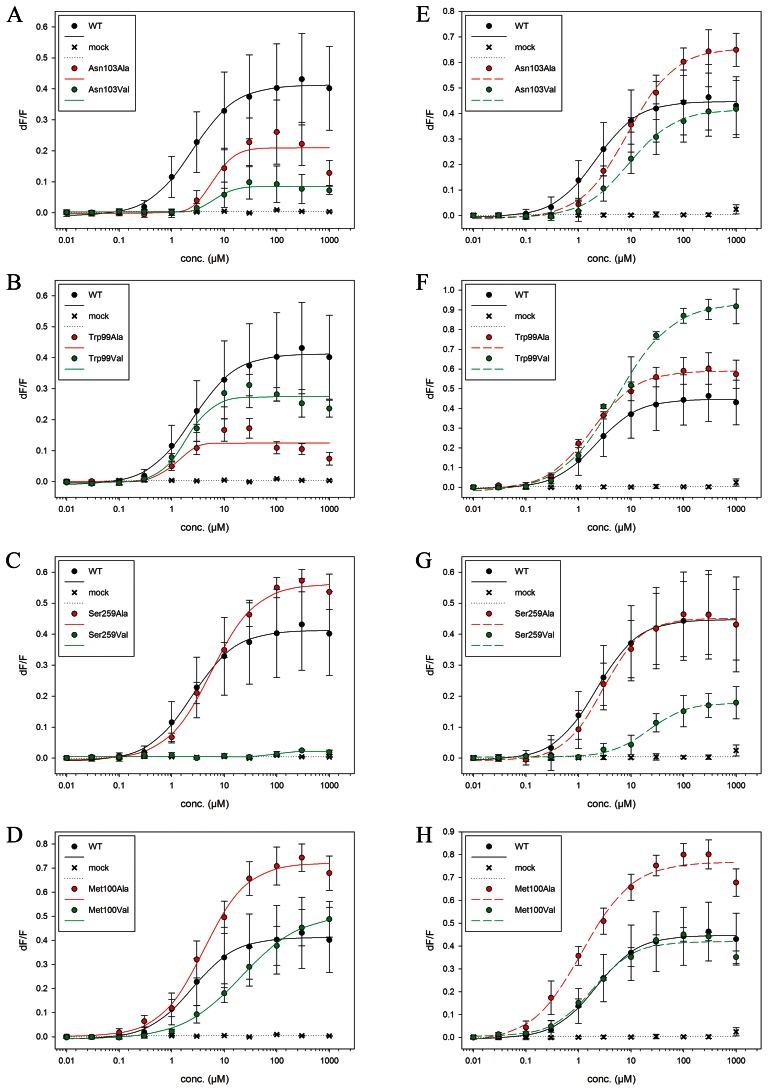
Dose-response curves. Dose-response curves of TAS2R38 wild type and mutants after stimulation with increasing PTC and PROP concentrations (0 to 1000 µM). Each point corresponds to the mean ± standard deviation. The mean is calculated from at least three independent experiments. **A–D)** PTC application, **E–H)** PROP application.

Functional assays have been reported by us for nine mutants of TAS2R38 in complex with PTC complex [9]. Those experiments were repeated here (Fig. 5A–D) and extended to the TAS2R38/PROP complex (Fig. 5E–H). The results turned out to be very similar for both agonists (Table 2, Fig. 5A–H): (i) The EC_50_ values of TAS2R38-Met100Ala (Fig. 5D), -Trp99Ala and -Trp99Val (Fig. 5B and F) are similar to those of WT. Those of the TAS2R38-Met100Val mutant turned out to be larger than WT (Fig. 5H). This may indicate that Met100 is close to the binding cavity and the presence of a valine residue may occlude the binding cavity. Consistently with these results, our simulations suggest that Met100 and Trp99 do not interact with the agonists but contribute to the shaping of the binding cavity. (ii) The EC_50_ values of TAS2R38-Asn103Ala and -Asn103Val (Fig. 5A and E) turn out to be larger than that of WT. Asn103 side-chain forms an H-bond with both agonists in our simulations. Hence, their mutations to Ala and Val should impair these interactions, consistently with the experimental results. (iii) The EC_50_ of TAS2R38-Ser259Val is larger than that of WT, whilst -Ser259Ala is similar to WT (Fig. 5C and G). Our simulations suggest that Ser259 is close to the agonists without any direct interaction. This is also consistent with experiment: the fact that the EC_50_ value increases for the Val mutants may be due to the presence of a bulkier residue in position 259, which with its higher steric hindrance could impair the binding. Instead, Ala, which is similar-in-size with Ser, is expected not to greatly affect the binding.

**Figure 6:**
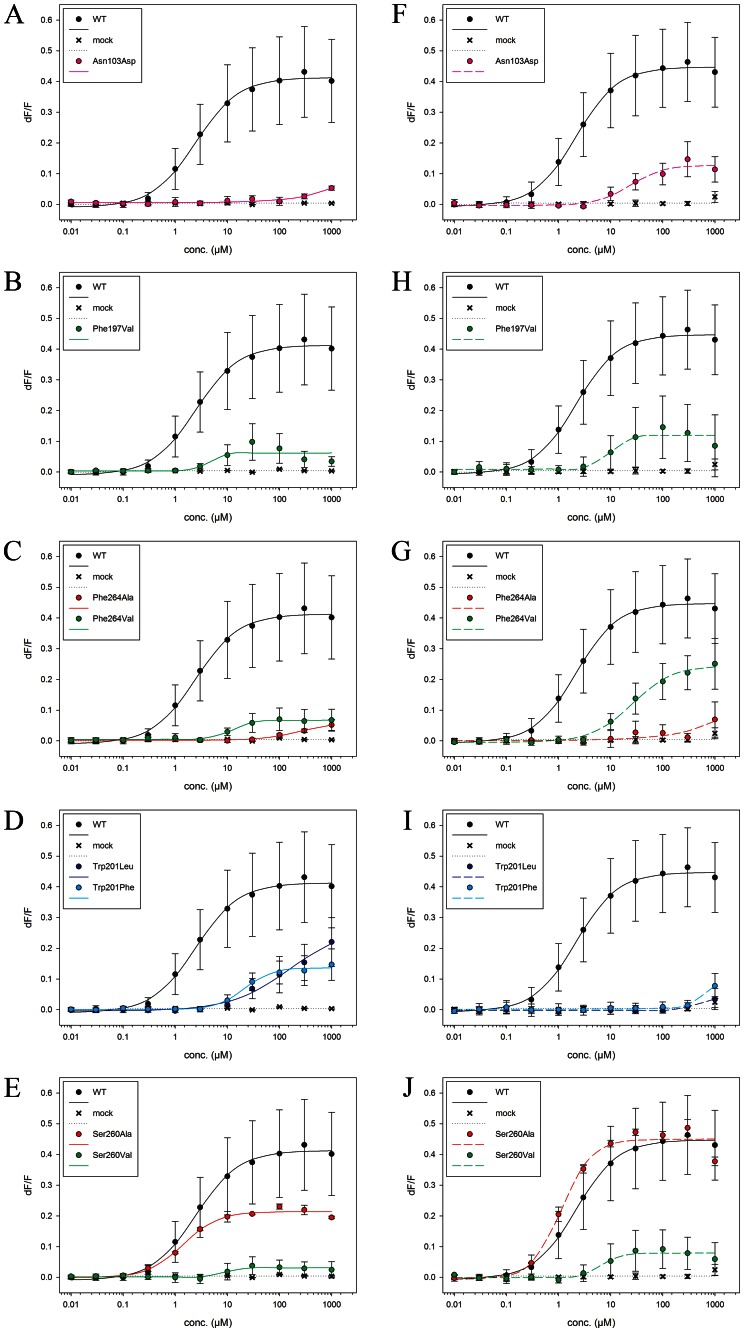
Dose-response curves. Dose-response curves of TAS2R38 wild type and mutants after stimulation with increasing PTC and PROP concentrations (0 to 1000 µM). Each point corresponds to the mean ± standard deviation. The mean is calculated from at least three independent experiments. **A-E)** PTC application, **F-J)** PROP application.

Next, we expressed new mutations, which may affect key receptor/agonists interactions and/or receptor binding cavity shape according to our MM/CG calculations (Table 1 and Fig. 3). These include: (i) The TAS2R38-Asn103Asp mutation, which should disrupt the Asn103/agonists sulphur atom H-bond. This causes a repulsive electrostatic interaction between Asp and the partially negatively charged sulphur atom of both agonists. This mutation should therefore severely impair binding (Fig. 6A and F and Table 2). Consistent with this hypothesis, the EC_50_ values of the mutant turn out to be much larger than those of the WT for both the agonists. (ii) The TAS2R38-Phe197Val mutation, which should affect Phe197 π-π stacking interactions with both agonists. Fairly consistently, the EC_50_ value of TAS2R38-Phe197Val turned out to be larger than that of WT upon PROP application (Fig. 6B and G). The EC_50_ value of the mutant turned out also to be larger, but not statistically significant, upon PTC application. This leads us to the suggestion that Phe197 forms stronger interactions with PROP than with PTC. (iii) The TAS2R38-Phe264Ala and -Phe264Val mutations, which should disrupt Phe264 π-π stacking interactions with both agonists. The mutation to Ala may further affect the binding cavity shape because of the small size of this residue. Consistent with this prediction, the EC_50_ of the mutants turned out to be larger than that of the WT (Fig. 6C and H). (iv) The TAS2R38-Trp201Leu mutation, should disrupt the Trp201 π-π stacking interactions with both agonists. Consistently, this mutation produced a severely impaired activation upon PTC application and an almost failed activation upon PROP application (Fig. 6D and I). (v) The TAS2R38-Trp201Phe mutant might affect the π-π stacking interactions (with both the agonists) due to the intrinsic difference between the two residues, and also disrupt the H-bond with Ser260 observed in the PTC adduct. The latter could play a role in shaping the binding cavity (Fig. 3). The EC_50_ of the adduct with PTC is larger than that of WT (Fig. 6D and I). Upon PROP application, we observe an almost complete loss of function of the receptor. (vi) The TAS2R38-Ser260 residues may play a role for shaping the binding cavity (it interacts with Trp201 in the adduct with PTC with as described in (v)) and/or for substrate binding (it interact with PROP). To address this issue, we investigate here the Val and Ala mutants in the position 260. We find that the EC_50_ values of Ser260Val mutant are larger than those of WT for both agonists, and those of TAS2R38-Ser260Ala are similar to those of WT (Fig. 6E and J). These data suggest that Ser260 participates in shaping the cavity and it may not be involved in interactions with the agonists.

## Discussion

We have presented a computational study of the TAS2R38 receptor in complex with its agonists PTC and PROP (Chart S1). We have used our recently developed hybrid MM/CG approach tailored for GPCRs to predict the structural determinants of the adducts. The method turned out to reproduce the interactions between the β2 AR with its ligand S-Car, even if the MM/CG calculations are based on a homology model. The subsequent MM/CG-based calculations of the hTAS2R38 complexes (based on structures obtained by homology modelling and molecular docking) have been tested against a pool of 22 molecular biology data (Table 2).

The calculations, validated by experimental evidence provided here and in ref. [9], show that the agonists interact Asn103, Phe197, Phe264 and Trp201. This prediction could not be made based on just our homology models and molecular docking structures (data not shown).

**Figure 7:**
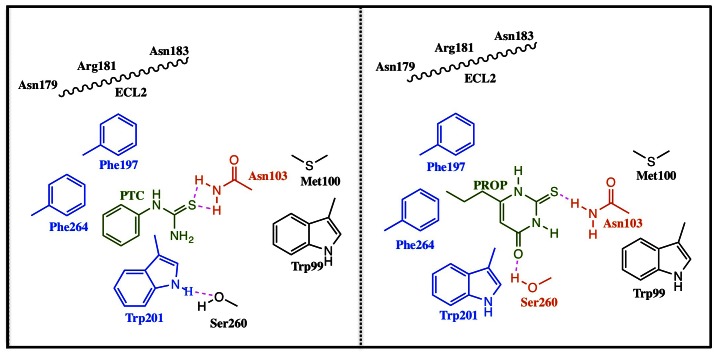
Agonists binding. Binding of PTC and PROP to the TAS2R38 bitter receptor as emerging from MM/CG simulations and experiments. Residues forming hydrophobic interactions and H-bonds with the agonists are indicated in blue and red, respectively. Residues shaping the cavities are in black color. The ECL2 loop does not interact directly with the agonists.

According to our modelling, Asn103 forms an H-bond with the agonists sulphur atom (Fig. 7). Asn103 corresponding position (3.36 according Ballesteros-Weinstein numbering [22]) play a role for ligand binding and/or for defining the binding cavity in other human bitter taste receptors, including hTAS2R46 [10]), hTAS2R31/R44, hTAS2R43 [23] and hTAS2R16 [11] (Table 3). It may do so in another GPCR, the 5-HT2A receptor [24]. In this receptor, the agonist 5-hydroxytryptamine, may form an H-bond with the side-chain of Ser3.36 (Ser159), consistent with the fact that the Ser3.36Ala mutation affects agonist binding.

**Table 3:** Analysis of the positions mutated in this work that were also mutated in other members of the bitter receptors family.

Position in TAS2R38	Effect in TAS2R38	Position in other TAS2 receptors	Effect in other TAS2 receptors
Ans103	Directly interacting with the agonist	Asn92 (R46) [10]	Interacting with agonist
		Asn89 (R16) [11]	H-bond with agonist
		Asn92 (R31/R44)) [23]	Direct interaction with agonist/shaping binding cavity
		Asn92 (R43/R61) [23]	Critical role in activation
Ser260	Interacting with the agonist or with Trp201	Phe240 (R16) [11]	Indirect participation in binding
Ser259	Not interacting but shaping the binding cavity	Tyr241 (R46) [10]	H-bond with agonist
Trp99	Not interacting but shaping binding cavity	Trp88 (R31/R44) [23]	Direct interaction with agonist/shaping binding cavity
Met100	Not interacting but shaping the binding cavity	Glu86 (R16) [11]	H-bond with agonist

In column 3, the receptor name is indicated between parentheses.

Several aromatic residues including the highly conserved Trp99 (Figure S1 and Table 3) and Phe264 shape the agonists binding cavity in both complexes. This supports the hypothesis [10] that the binding cavities of bitter taste receptors are similar for different agonists. Some of them might do so even across different receptors of the family. We suggest this based on the fact that Trp99 corresponding position in hTAS2R31 (Trp88) also shapes the binding cavity in that receptor [23] (Table 3).

Ser260 may contribute to shape the binding cavity of the receptor. The corresponding position of Ser260 (6.52 according to Ballesteros-Weinstein numbering [22]) might form hydrophobic interactions with salicin agonists of hTAS2R16 (Phe240 in [11]) and an H-bond with strychnine agonist in hTAS2R46 (Tyr241 in [10]). Hence, this position might be important for receptor function across several members of the bitter taste family.

The ECL2 loop consists of non-conserved residues across the family (Fig. S1) [25]. It is located relatively far from the binding site. It does not form any interaction with the agonists. [19], [26]. This is a common feature of all GPCRs for which X-ray structural information is available other than rhodopsin [25], [27]. Because ECL2 displays a highly conserved N-glycosylation site [26] one has to exercise care for any implications of these findings for the function of the receptor in in vivo conditions.

Responses to PROP and PTC of the same mutant variants sometimes are different (Table 2) in both maximal signal amplitude and in EC_50_ values. Indeed, while TAS2R38-Trp99 mutant variants show higher maximal amplitude upon PROP application than upon PTC application, TAS2R38-Met100, Phe197 and Ser260 show different behaviour upon PROP or PTC application, indicating a different role of the residues in binding of a specific ligand. Thus, our data support the hypothesis [10] that different agonists bind to similar but not identical binding cavities. Probably, some crucial residues are involved in binding of several different agonists, i.e. Asn103, and they also are quite well conserved in the family, whereas other different residues are directly interacting with different agonist. Hence, although several residues (Asn103, Phe197, Trp201 and Phe264) interact with both agonists, our results point out that the different response of the two agonists is due to a small but still significant difference in the binding cavities.

### Receptor Activation

Differences in maximal signal amplitude (MSAs) of the mutants investigated here may reflect differences on the receptor activation mechanism. In this section, we discuss changes in MSAs, along with bioinformatics arguments, to gain insights into the possible role of receptors residues for its activation. We start our discussion with two highly conserved residues, Trp99 and Asn103 (Fig. S1). The first, according to our modelling, does not interact directly to the agonists, whilst the second does (Fig. 7). The MSAs of the mutants involving the two positions differ from those of WT for both agonists (Fig. 4–6). Hence, we suggest for these positions to be important for the activation of this receptor and, because of the high conservation of these residues, maybe for other members of the family. At present, it is difficult to suggest any mechanistic role for these residues. Phe197, Trp201 (on TM5) and Phe264 (on TM6) are instead highly variable residues in the family (Fig. S1). The MSAs of their mutants are very different than those of WT, suggesting a role for receptor activation also for these residues. We suggest that some of the mutations may affect stacking interactions formed by these aromatic residues, which in turn are important for activation with the PROP and PTC agonists.

We next focus on the TAS2R38 naturally polymorphic positions (49, 262, and 296), which lead to impairment of receptor activity [6], [7]. Ala262 and Val296 are located in TM6 and TM7, respectively. Val296 is far away from the binding cavity and Ala262, although closer is not pointing to it or interacting with the agonists (Fig. S4). Hence, differently to what claimed in ref [28], residues in the two positions, Ala262 and Val296, might be involved in the transduction mechanism rather than in ligand binding. Position 49 in the models is located in a loop region (IC1) far away form the putative binding cavity and due to the difficulties in loop modelling at the present stage, it is impossible to hypothesize a role in the activation mechanisms of the receptor.

In conclusion the protocol described here, which includes MM/CG simulations on homology models and experiments, could be applied to different members of the bitter taste receptors as well as from the GPCR superfamily. The code, along with the MM/CG trajectories as well as the homology models resulting from this research, are freely available upon request.

## Materials and Methods

### Biocomputing

We generated structural models of TAS2R38 in the following steps, as in [9]: (i) Identifying all human bitter taste receptors from the Uniprot database (http://www.uniprot.org/), (ii) aligning the sequence of the proteins identified in (i) using Promals [29] (Fig. S1, SI). (iii) The aligned sequences from point (ii) were funnelled to the HHpred web server (http://toolkit.tuebingen.mpg.de/hhpred). (iv) Retrieving the sequences of the templates and their corresponding structures. Some of them have been already included in ref. [9] (highlighted in yellow in Table S1) (v) Constructing 200 models of TAS2R38 based on the alignment in (iii) and the 3D structures of the templates in (iv), that is, all the GPCRs with known structure. The Modeller9v3 program was used [30]. The representative of the two most populated clusters extracted from the model structural ensemble (**A** and **B**, see Results Section) turned out to represent 70% of the conformational ensemble, according to the clustering algorithm of ref. [31]. They were very similar one to the other except for the extracellular loop ECL2. (vi) Generating models of the PTC/TAS2R38 complex in **A** conformation (PTC/**A** hereafter), PTC/TAS2R38 in **B** conformation (PTC/**B**), PROP/TAS2R38 in **A** conformation (PROP/**A**), PROP/TAS2R38 in **B** conformation (PROP/**B**). We docked phenylthiocarbamide (PTC) and propylthiouracil (PROP, Fig. 3) on **A** and **B** using the Haddock program [32]. Asn103 binds to PTC, and hence probably to PROP, because PROP is structurally not too dissimilar to PTC (Fig. 3). Hence, it was defined as an ‘active’ residue in the docking procedure of PTC/**A**, PTC/**B**, PROP/**A**, PROP/**B** [32]. Trp99, Met100, Ser259 were shown to shape the binding cavity [9]. They were defined as ‘passive’ residues in PTC/**A**, PTC/**B**, PROP/**A**, PROP/**B**. The 200 top complexes for each of those (according to the Haddock’s scoring function [32]) underwent energy minimization in explicit water, using Haddock [32]. The resulting structures were clustered using the algorithm in ref. [31]. (vii) The four top complexes were chosen as the ones satisfying all the active and passive restraints and with lower energy values as from Haddock scoring function. They underwent two replicas of 0.6 µs molecular mechanics/coarse-grained MM/CG molecular dynamics simulations [14], [15]. The complexes were split in a MM part, which includes the GPCR agonist (or inverse agonist) and the residues in the binding cavity (Fig. S2, SI), in a CG part, containing the protein frame and in an interface region (I), defined between the MM and CG regions. Hydration at the active site was accounted by including a 15 Å droplet of water molecules around the MM region. The presence of the lipid bilayer was taken into account introducing a wall located at 2.0 Å from the proteins C*α* atoms. In particular, β2 AR.S-Car and TAS2R38 were encapsulated in a ∼31 Å thick implicit membrane. Two planar walls coincide with the height of the lipid heads, two hemispheric walls cap the extracellular and cytoplasmic ends of the protein. The last wall (‘membrane wall’) follows the initial shape of the interface between protein and membrane. Details on the MM/CG methodology can be found in reference [14]. The proteins were partitioned in the MM and GC regions. For β2-AR.S-Car, the first consists of residues 79–82, 86, 109 to 118, 164–165, 193–195, 199–208, 282, 286, 289–290, 293, 308, 311–316, and the second is given by the rest of the protein. For TAS2R38, the first consists in residues 14–23, 70–101, 103–104, 151–164, 187, 189, 193–204, 258–268 and 277–287, and the second the rest of the protein. The MM and the I regions were described with the GROMOS 96 force field [33]. RESP charges [34] were derived for the agonists (PTC and PROP), using structures optimized with HF-6-31G*, obtained with Gaussian03 [33]. Bonded parameters were obtained using the PRODRG server [35]. Water is described with the SPC force field [36]. The CG part is described using a Go-like potential [14], [17]. We used a cutoff of 16 Å for electrostatics, Van der Waals and Go-like interactions. Residues included in the I region are determined as the residues at a distance shorter or equal than 6 Å from the MM boundary. The SHAKE algorithm was used to keep fixed the distance of bonds containing hydrogen(s) [37]. The simulations were performed at a constant temperature (300 K). The thermostat in stochastic dynamics was controlled by setting the inverse friction constant at a value of 0.4 ps. Once obtained, the trajectories of each replica corresponding to each of the complexes were joint and analysed performing a clustering analysis. For this purpose, all the protein backbone was aligned and later the conformations were clustered according to the position of the ligand. A 1 Å cut-off was used to group the structures in the same cluster.

### Experiments

#### Site-directed mutagenesis

TAS2R38 mutants were obtained by site-directed mutagenesis PCR using mutagenesis overlapping primers and TAS2R38-PAV variant cDNA cloned into a pcDNA5/FRT plasmid (Invitrogen) as template. For the list of used oligonucleotides, refer to Table S2. The subsequent PCR-mediated recombination using CMV forward primer, located upstream of the cDNA sequence, and BGH reverse primer, located downstream of the cDNA sequence was performed to join the overlapping mutant fragments. The mutant cDNA sequences were digested with EcoRI and NotI restriction enzymes, to be cloned into a previously digested pcDNA5/FRT. The plasmid presented an amino terminal export tag corresponding to the first 45 amino acids of rat somatostatin receptor 3 and a carboxy terminal HSV tag [6], [38], [39]. The resulting mutant cDNA constructs were sequenced to confirm their integrity [39].

### Immunocytochemistry

The different mutant variants, as well as the TAS2R38-PAV variant, were transiently transfected with Lipofectamine2000 (Invitrogen) in HEK 293T cells stably expressing the chimeric G protein subunit Gα16gust44, very effective in coupling with bitter taste receptors [40] [41]. HEK 293T cells were seeded on poly-D-lysine coated coverslips and transfected with the different TAS2R38 variants. Cells were washed with 37°C warm PBS 24 hr after transfection and incubated on ice for 1 hr. Later, they were incubated on ice with biotin-labelled Concanavalin A (Molecular Probes) for plasma membrane staining and fixed and permeabilised with aceton-methanol 1∶1 solution. Blocking was done using 5% horse serum in PBS and antibody incubation was performed over night at 4°C with 1∶15000 mouse anti-HSV primary antibody (Novagen). Secondary antibody incubation included both 1∶1000 Streptavidin Alexa Fluor633 to label plasma membrane and 1∶1000 Alexa488-conjugated anti-mouse IgG (Molecular Probes) to label receptors (in 5% horse serum PBS), for 1 hr at room temperature. Coverslips were mounted in Dako mounting medium and analysed with a Leica confocal microscope [6] [39].

### Calcium Imaging Experiments

The different mutant variants, as well as the TAS2R38-PAV variant, were transiently transfected with Lipofectamine2000 (Invitrogen) in HEK 293T cells stably expressing the chimeric G protein subunit Gα16gust44. [3], [40] 24 hours after transfection, cells were loaded with Ca^2+^ sensitive Fluo4-AM dye, washed 3 times in C1 buffer (130 mM NaCl, 5 mM KCl, 10 mM Na-Hepes, 2 mM CaCl_2_ and 10 m M Glucose, pH 7.4) and changes in intracellular Ca^2+^ concentration upon agonist solution application were recorded, at least 3 times independently for each mutant variant, using a fluorometric imaging plate reader FLIPR^TETRA^ (Molecular Devices). Agonists were dissolved into C1 buffer in a range of 0–1000 µM concentration. Experiments with previously reported mutant variants upon PTC application [9] were repeated for the present article in order to obtain fully comparable results. Positive (TAS2R38-PAV variant) and negative (mock transfected) controls were performed.

## Supporting Information

Figure S1 **Alignment of the human bitter taste receptors sequences family.** These were retrieved from the Uniprot database (http://www.uniprot.org/). The multiple sequence alignment was carried out using the program Promals [29]. Green columns correspond to the naturally polymorphic residues Pro49, Ala262 and Val296. Red columns comprise residues: Trp99, Met100, and Ser260. Blue columns indicate residues: Asn103, Phe197, Trp201, Ser259 and Phe264. Finally the grey region indicates the poorly conserved ECL2.
(TIF)

Figure S2 **Two independent MM/CG simulations were carried out for PTC/A, PTC/B, PROP/A and PROP/B.** Here we plot the RMSD of the Cα atoms of the four complexes as a function of time for both simulations (red and black continuous curves). Part of the ECL2 (residues 168 to 178) is very mobile in one of the simulations of PROP/**B**, causing an increase of the RMSD values (continuous red curve). However, it does not interact at all with the agonist. The RMSDs of the protein excluding residues 168 to 178 is indeed not too dissimilar to that of the overall RMSD (dotted red line).
(TIF)

Figure S3 **Largest cavities of models A and B identified using Fpocket** **[42]**, **[43]** **.**
(TIFF)

Figure S4 **Location of three major naturally polymorphic positions.** Residues Ala262(blue), Val296(red) and Pro49(green) in the central structure of the principal clusters found in the MM/CG simulation of PTC/**B** (left) and PROP/**B** (right). Val296 and Pro49 belong to the CG region, thus only the Cα atom is shown. The positions of PTC and PROP agonists after the docking are shown in cyan and yellow, respectively.
(TIFF)

Table S1 **List of the GPCRs for which X-ray structures are available.** Their corresponding PDB codes, co-crystallized ligands, species and resolution values are indicated. The structures used in our previous work are highlighted in yellow [9].
(DOC)

Table S2 **List of oligonucleotides used in site-directed mutagenesis.**
(DOC)

Text S1 **Supporting information.** Ligand binding cavity volume calculations (calculated using the program FPocket [42], [43]). Molecular docking results
(DOC)

Chart S1 **Chemical structures of the TAS2R38 ligands used in the present work.**
(TIF)
