# Supplementary figures and images for: Coarse-Grained/Molecular Mechanics of the TAS2R38 Bitter Taste Receptor: Experimentally-Validated Detailed Structural Prediction of Agonist Binding
Source: PLoS One. 2013 May 31;8(5):e64675. doi: 10.1371/journal.pone.0064675 (PMC3669430; doi:10.1371/journal.pone.0064675)

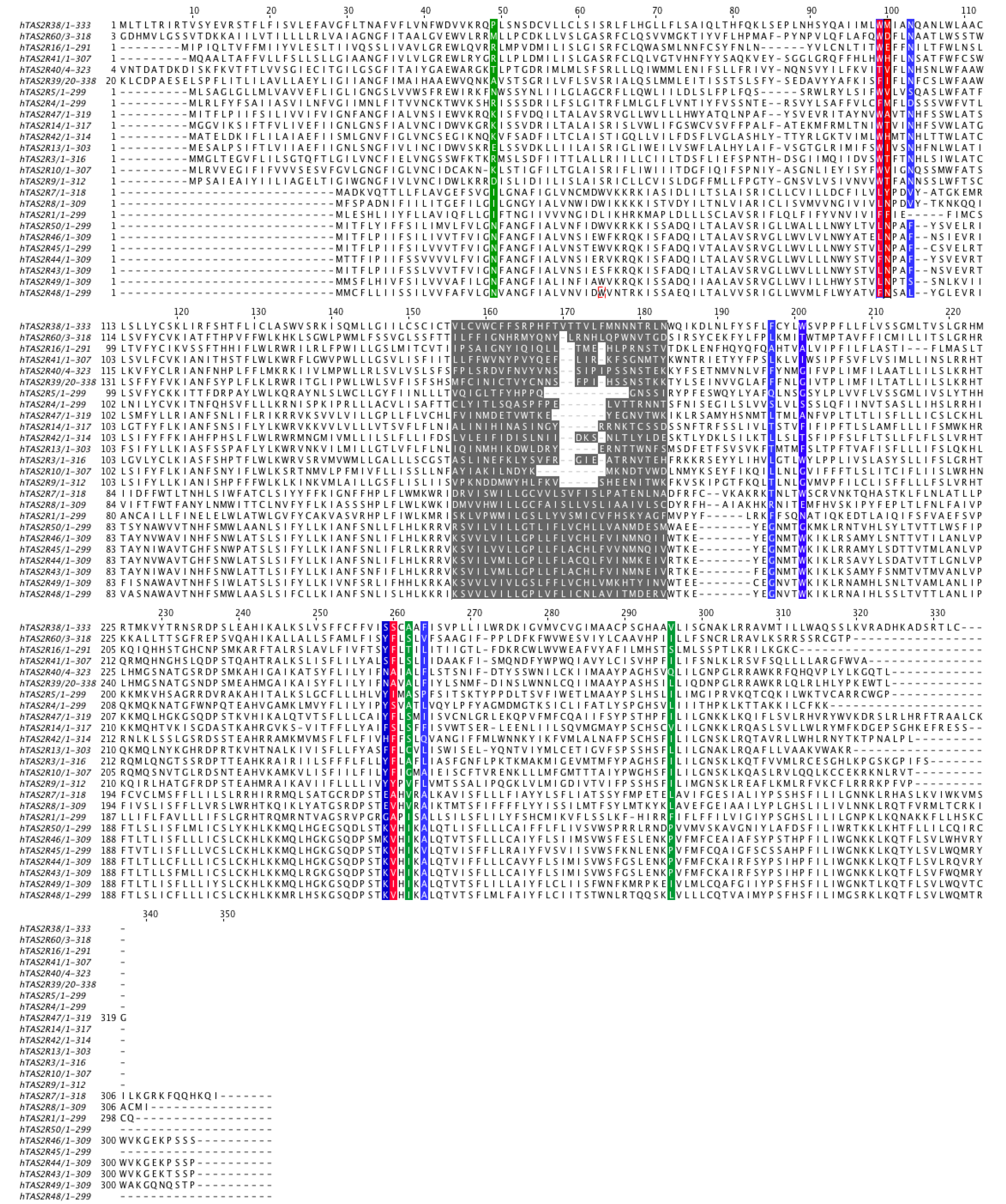

Supplement: Figure S1 — Alignment of the human bitter taste receptors sequences family. These were retrieved from the Uniprot database (http://www.uniprot.org/). The multiple sequence alignment was carried out using the program Promals [29]. Green columns correspond to the naturally polymorphic residues Pro49, Ala262 and Val296. Red columns comprise residues: Trp99, Met100, and Ser260. Blue columns indicate residues: Asn103, Phe197, Trp201, Ser259 and Phe264. Finally the grey region indicates the poorly conserved ECL2. (TIF) [file pone.0064675.s001.tif]

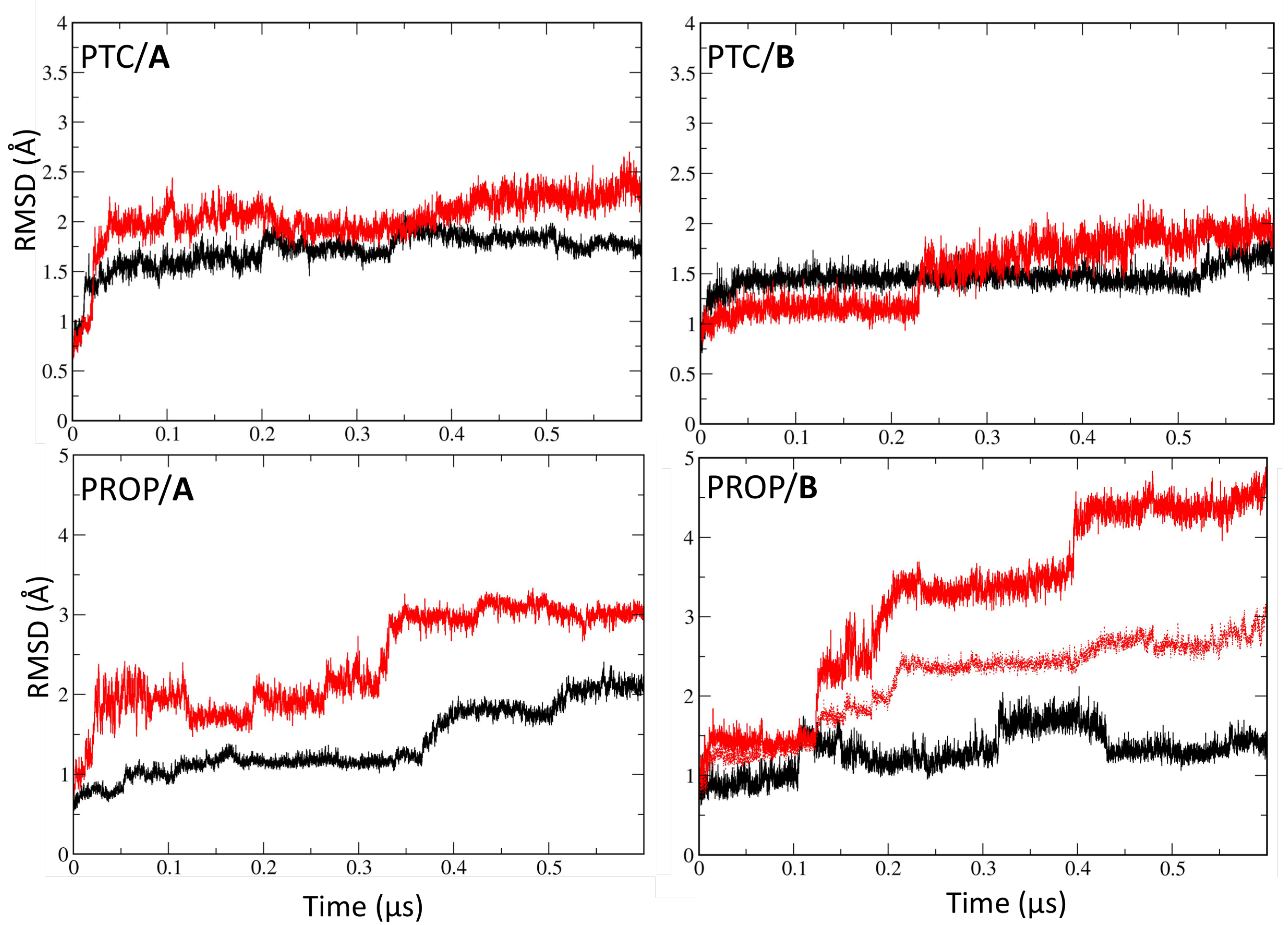

Supplement: Figure S2 — Two independent MM/CG simulations were carried out for PTC/A, PTC/B, PROP/A and PROP/B. Here we plot the RMSD of the Cα atoms of the four complexes as a function of time for both simulations (red and black continuous curves). Part of the ECL2 (residues 168 to 178) is very mobile in one of the simulations of PROP/B, causing an increase of the RMSD values (continuous red curve). However, it does not interact at all with the agonist. The RMSDs of the protein excluding residues 168 to 178 is indeed not too dissimilar to that of the overall RMSD (dotted red line). (TIF) [file pone.0064675.s002.tif]

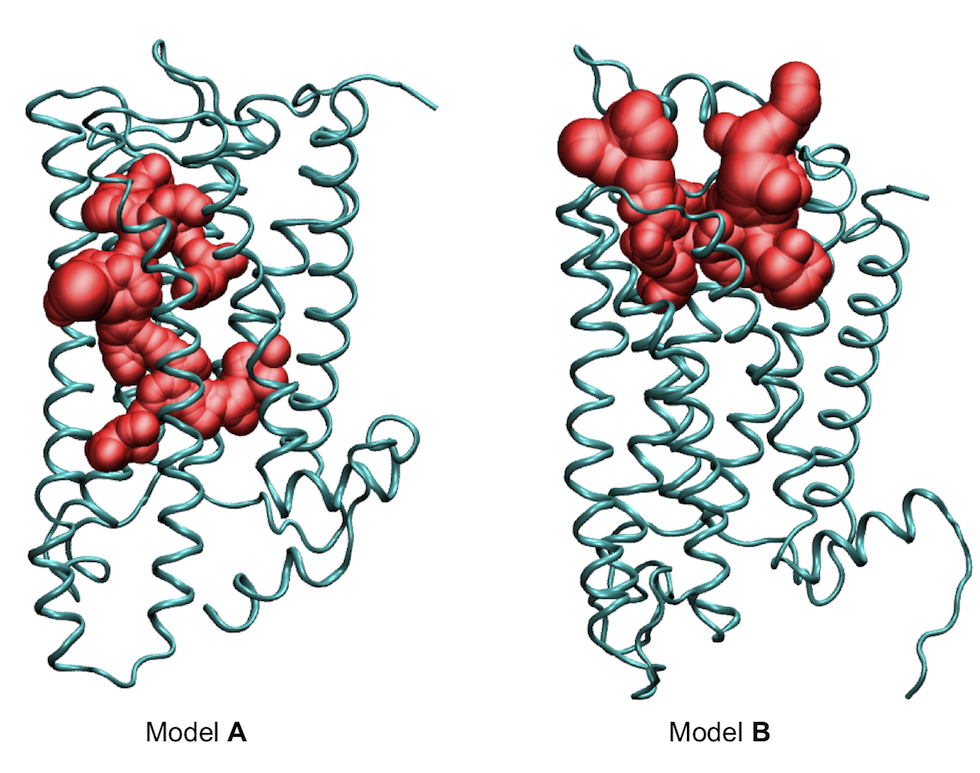

Supplement: Figure S3 — Largest cavities of models A and B identified using Fpocket [42] , [43] . (TIFF) [file pone.0064675.s003.tiff]

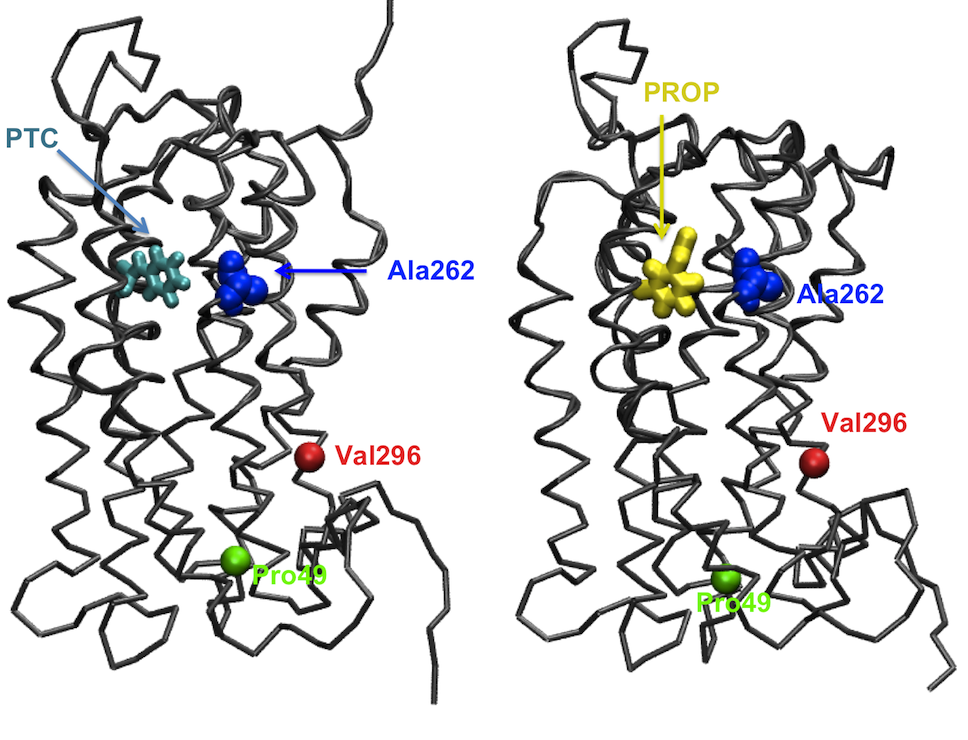

Supplement: Figure S4 — Location of three major naturally polymorphic positions. Residues Ala262(blue), Val296(red) and Pro49(green) in the central structure of the principal clusters found in the MM/CG simulation of PTC/B (left) and PROP/B (right). Val296 and Pro49 belong to the CG region, thus only the Cα atom is shown. The positions of PTC and PROP agonists after the docking are shown in cyan and yellow, respectively. (TIFF) [file pone.0064675.s004.tiff]

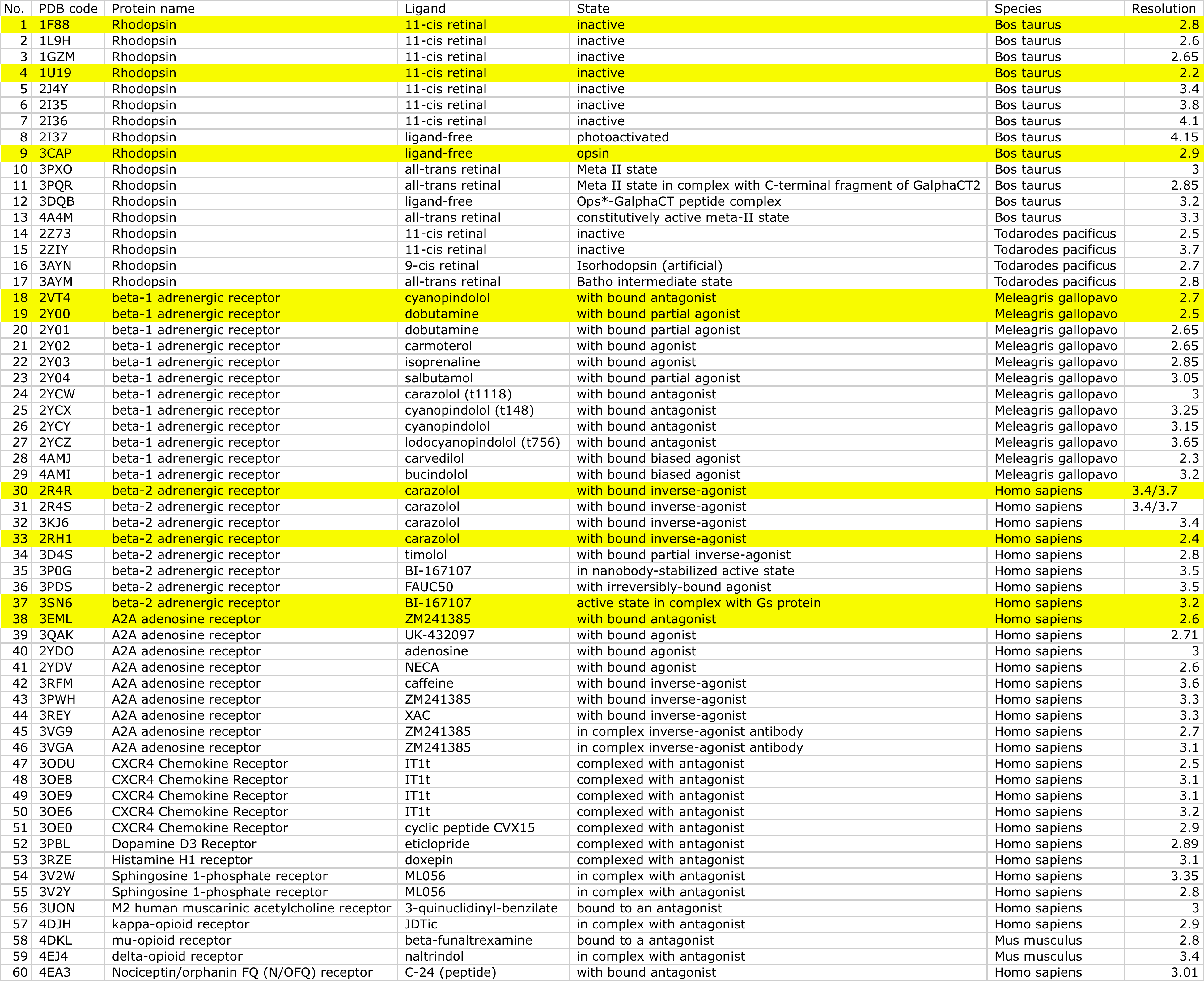

Supplement: Table S1 — List of the GPCRs for which X-ray structures are available. Their corresponding PDB codes, co-crystallized ligands, species and resolution values are indicated. The structures used in our previous work are highlighted in yellow [9]. (DOC) [file pone.0064675.s005.doc]

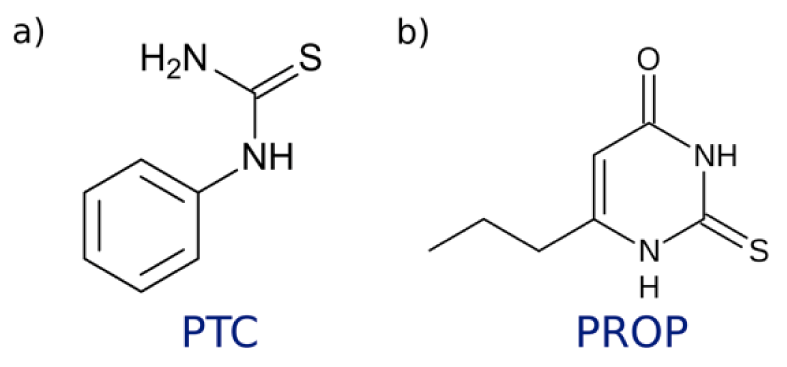

Supplement: Chart S1 — Chemical structures of the TAS2R38 ligands used in the present work. (TIF) [file pone.0064675.s008.tif]
