# Supplementary material for: Coarse-Grained/Molecular Mechanics of the TAS2R38 Bitter Taste Receptor: Experimentally-Validated Detailed Structural Prediction of Agonist Binding
Source: PLoS One. 2013 May 31;8(5):e64675. doi: 10.1371/journal.pone.0064675 (PMC3669430; doi:10.1371/journal.pone.0064675)
Supplement: Table S2 — List of oligonucleotides used in site-directed mutagenesis. (DOC) [file pone.0064675.s006.doc]

| **Oligonucl. name** | **Sequence** |
| --- | --- |
| W99A Forward  W99A Reverse | GCTACCAAGCCATCATCATGCTAGCGATGATTGCAAACCAAGC  GCTTGGTTTGCAATCATCGCTAGCATGATGATGGCTTGGTAGC |
| W99V Forward  W99V Reverse | GCTACCAAGCCATCATCATGCTAGTGATGATTGCAAACCAAGC  GCTTGGTTTGCAATCATCACTAGCATGATGATGGCTTGGTAGC |
| M100A Forward  M100A Reverse | GCCATCATCATGCTATGGGCGATTGCAAACCAAGCCAACC  GGTTGGCTTGGTTTGCAATCGCCCATAGCATGATGATGGC |
| M100V Forward  M100V Reverse | GCCATCATCATGCTATGGGTGATTGCAAACCAAGCCAACC  GGTTGGCTTGGTTTGCAATCACCCATAGCATGATGATGGC |
| N103A Forward  N103A Reverse | CCATCATCATGCTATGGATGATTGCAGCCCAAGCCAACCTCTGG  CCAGAGGTTGGCTTGGGCTGCAATCATCCATAGCATGATGATGG |
| N103V Forward  N103V Reverse | CCATCATCATGCTATGGATGATTGCAGTCCAAGCCAACCTCTGG  CCAGAGGTTGGCTTGGACTGCAATCATCCATAGCATGATGATGG |
| N103D Forward  N103D Reverse | GCTATGGATGATTGCAGACCAAGCCAACCTCTGG  CCAGAGGTTGGCTTGGTCTGCAATCATCCATAGC |
| N179A Forward  N179A Reverse | GCTATTCATGAATAACGCTACAAGGCTCAACTGGC  GCCAGTTGAGCCTTGTAGCGTTATTCATGAATAGC |
| N179V Forward  N179V Reverse | GCTATTCATGAATAACGTTACAAGGCTCAACTGGC  GCCAGTTGAGCCTTGTAACGTTATTCATGAATAGC |
| R181A Forward  R181A Reverse | CATGAATAACAATACAGCGCTCAACTGGCAG  CTGCCAGTTGAGCGCTGTATTGTTATTCATG |
| R181V Forward  R181V Reverse | CATGAATAACAATACAGTGCTCAACTGGCAG  CTGCCAGTTGAGCACTGTATTGTTATTCATG |
| N183A Forward  N183A Reverse | CAATACAAGGCTCGCCTGGCAGATTAAAG  CTTTAATCTGCCAGGCGAGCCTTGTATTG |
| N183V Forward  N183V Reverse | CAATACAAGGCTCGTCTGGCAGATTAAAG  CTTTAATCTGCCAGACGAGCCTTGTATTG |
| F197V Forward  F197V Reverse | CAATTTATTTTATTCCTTTCTCGTCTGCTATCTGTGGTCTGTGCC  GGCACAGACCACAGATAGCAGACGAGAAAGGAATAAAATAAATTG |
| W201L Forward  W201L Reverse | CCTTTCTCTTCTGCTATCTGTTGTCTGTGCCTCCTTTCC  GGAAAGGAGGCACAGACAACAGATAGCAGAAGAGAAAGG |
| W201F Forward  W201F Reverse | CTCTTCTGCTATCTGTTCTCTGTGCCTCCTTTCC  GGAAAGGAGGCACAGAGAACAGATAGCAGAAGAG |
| F252V Forward  F252V Reverse | CCTCAAGTCTCTTGTCTCCGTTTTCTGCTTCTTTGTGATATCATCC  GGATGATATCACAAAGAAGCAGAAAACGGAGACAAGAGACTTGAGG |
| F256V Forward  F256V Reverse | CTCTTGTCTCCTTTTTCTGCTTCGTTGTGATATCATCCTGTG  CACAGGATGATATCACAACGAAGCAGAAAAAGGAGACAAGAG |
| S259A Forward  S259A Reverse | CCTTTTTCTGCTTCTTTGTGATAGCATCCTGTGCTGCCTTCATC  GATGAAGGCAGCACAGGATGCTATCACAAAGAAGCAGAAAAAGG |
| S259V Forward  S259V Reverse | CCTTTTTCTGCTTCTTTGTGATAGTATCCTGTGCTGCCTTCATC  GATGAAGGCAGCACAGGATACTATCACAAAGAAGCAGAAAAAGG |
| S260A Forward  S260A Reverse | GCTTCTTTGTGATATCAGCCTGTGCTGCCTTCATC  GATGAAGGCAGCACAGGCTGATATCACAAAGAAGC |
| S260V Forward  S260V Reverse | CCTTTTTCTGCTTCTTTGTGATATCAGTCTGTGCTGCCTTCATC  GATGAAGGCAGCACAGACTGATATCACAAAGAAGCAGAAAAAGG |
| F264A Forward  F264A Reverse | CATCCTGTGCTGCCGCCATCTCTGTGCCC  GGGCACAGAGATGGCGGCAGCACAGGATG |
| F264V Forward  F264V Reverse | CATCCTGTGCTGCCGTCATCTCTGTGCCC  GGGCACAGAGATGACGGCAGCACAGGATG |
