# Supplementary material for: Coarse-Grained/Molecular Mechanics of the TAS2R38 Bitter Taste Receptor: Experimentally-Validated Detailed Structural Prediction of Agonist Binding
Source: PLoS One. 2013 May 31;8(5):e64675. doi: 10.1371/journal.pone.0064675 (PMC3669430; doi:10.1371/journal.pone.0064675)
Supplement: Text S1 — Supporting information. Ligand binding cavity volume calculations (calculated using the program FPocket [42], [43]). Molecular docking results (DOC) [file pone.0064675.s007.doc]

**SUPPORTING INFORMATION**

**Coarse-Grained/Molecular Mechanics of the TAS2R38 Bitter Taste Receptor: Experimentally-Validated Detailed Structural Prediction of Agonist Binding**

**AUTHORS**

Alessandro Marchiori^1,3,6,†^, Luciana Capece^2,3,6,†^, Alejandro Giorgetti^3,4,†^, Paolo Gasparini^5^, Maik Behrens^6^, Paolo Carloni^3,7,*^ and Wolfgang Meyerhof^6^

^1^International school for advanced studies (SISSA-ISAS), Neuroscience sector, via Bonomea 265, Trieste, Italy. ^2^International Centre for Genetic Engineering and Biotechnology, Trieste, Italy.^3^ Computational Biophysics, German Research School for Simulation Sciences, D-52425 Juelich, Germany. ^4^Dept of Biotechnology, University of Verona, Verona, Italy. ^5^ Institute for Maternal and Child Health - IRCCS “Burlo Garofolo”,Trieste, Italy. ^6^ Department of Molecular Genetics, German Institute of Human Nutrition Potsdam-Rehbruecke (DIfE), D-14558 Nuthetal, Germany. ^6^Institute for Advanced Simulation IAS-5, Computational Biomedicine, Forschungszentrum Juelich, D-52425 Juelich, Germany.

^*^To whom correspondence should be addressed.

**^†^These authors contributed equally.**

***1. Ligand binding cavity volume calculations****.* We calculated the volume of the ligand binding cavity for **A** and **B** using the FPocket online module [1, 2]. We obtained a total of 27 cavities for the model **A** and 28 cavities for model **B**. The larger cavities for both models were those corresponding to the ligand binding pocket. They have a volume of 1842 Å^3^ and 2929 Å^3^ for models **A** and **B**, respectively. The volume of the cavities of all the GPCRs with known structures range from 756 Å^3^ for the rhodopsin (1U19) to 2831 Å^3^ of β2 adrenergic receptor (1RH1) as calculated with FPocket [1, 2]. Interestingly, while the cavity of model **B** is solvent accessible that of model **A** is not (Fig. S3). However, agonists could still enter the cavity in **A** provided that ECL2 experiences structural fluctuations.

***2. Molecular docking results****.* The most populated clusters of PTC/**A**, PTC/**B,** PROP/**A,** PROB/**B** are formed by 120, 100, 80, 90 structures out of 200, respectively. They are those considered to MM/CG simulations.

In PTC/**A**, the agonist forms hydrophobic interactions with Phe197 ring. PTC sulphur atom form an H-bond with Ser260-OH group. In PTC/**B,** the agonist forms hydrophobic interactions with Phe197, Trp201, Trp99, Phe264. It also forms the same H-bond as PTC/**A**. In PROP/**A** the agonist sulphur atom forms an H-bond with the side-chain of Asn103. The NH group adjacent to the sulphur side-chain (Chart I main text) forms an H-bond with the carbonyl group of Asn103. The second NH group of the agonist forms an H-bond with Ser260 hydroxyl group. In PROP/**B**, the agonist's aromatic oxygen atom forms an H-bond with Asn103 side-chain amino group. Its NH group forms an H-bond with Asn103 side-chain carbonyl group. The agonist sulphur atom forms an H-bond with Gln104 side-chain.

**REFERENCES**

1. Schmidtke P, Le Guilloux V, Maupetit J, Tuffery P (2010) fpocket: online tools for protein ensemble pocket detection and tracking. Nucleic Acids Res 38: W582-589.

2. Le Guilloux V, Schmidtke P, Tuffery P (2009) Fpocket: an open source platform for ligand pocket detection. BMC Bioinformatics 10: 168.
